# Adeno-associated virus-mediated intraprostatic suppression of *MIR375* inhibits tumor progression in the TRAMP mouse model of prostate cancer

**DOI:** 10.1016/j.gendis.2023.101182

**Published:** 2023-11-23

**Authors:** Xianyanling Yi, Jin Li, Zeyu Han, Tianyi Zhang, Dazhou Liao, Jia You, Jianzhong Ai

**Affiliations:** Department of Urology/Institute of Urology, West China Hospital, Sichuan University, Chengdu, Sichuan 610041, China

The treatment of prostate cancer (PCa) needs to be improved.^1^ Micro-RNAs (miRNAs) are a subtype of non-coding, single-stranded RNAs that influence cellular survival and death by modulating mRNAs. Among these miRNAs, *MIR3*75 has a critical role in the regulation of tumorigenesis^2^ and holds promise as a novel therapeutic target for future PCa treatment. Recombinant adeno-associated virus (rAAV) exhibits non-pathogenicity, low-grade inflammation, and robust and long-lasting expressions of target genes. We have previously described the rAAV9 as a valid vector to transfer target miRNAs and genes,^3^ so rAAV9 could be used as a vector to deliver *MIR375* into the mouse prostate and PCa cells.

We initially obtained relative expression of *MIR375* in PCa from The Cancer Genome Atlas (TCGA) database (http://cancergenome.nih. gov/), and the expression level of *MIR375* was compared between tumor and normal tissues. As depicted in Figure 1A, tumor tissues exhibited a significantly higher expression level of *MIR375* than normal tissues. Subsequently, transgenic adenocarcinoma mouse prostate (TRAMP) models of different ages were sacrificed, and prostates were harvested. Total RNA was extracted and *Mir375* expression was analyzed via quantitative real-time PCR. A remarkable increase in the expression level of *Mir375* in TRAMP mice in comparison to wild-type mice. Furthermore, it was observed that the expression level of *Mir375* in TRAMP mice increased with age (Fig. 1B). Then, to assay for *MIR375* activity, we constructed a dual-gene reporter vector. The data showed that *LacZ/Fluc* reporter activity was significantly down-regulated by *MIR375* (Fig. S1A). It indicated that *MIR375* could exert its regulatory function via the vector pAAVsc CB PI- miR-375-Gluc. Moreover, tough decoy (TuD) antisense RNA was used, an efficient and specific inhibitor of miRNA, and the *LacZ/Fluc* reporter activity was restored (Fig. 1C). Thus, the expression of *MIR375* was reduced using TuD in subsequent experiments. The effect of *MIR375* on PC3 cell apoptosis was examined with flow cytometry, and the inhibition of MIR375 led to a significant increase in apoptotic cell death, encompassing both early and late apoptosis stages (Fig. 1D; Fig. S1B). Cell viability was detected by CCK8 assay. According to Figure 1E, the proliferation of PC3 cells was attenuated upon *MIR375* inhibition. Furthermore, the scratch experiment showed that the scratch width in the TuD-MIR375 group was wider than that of the control group after 24 h and 48 h (Fig. 1F). These findings collectively suggested that *MIR375* promotes the proliferation and migration of PC3 cells.

**Figure 1:**
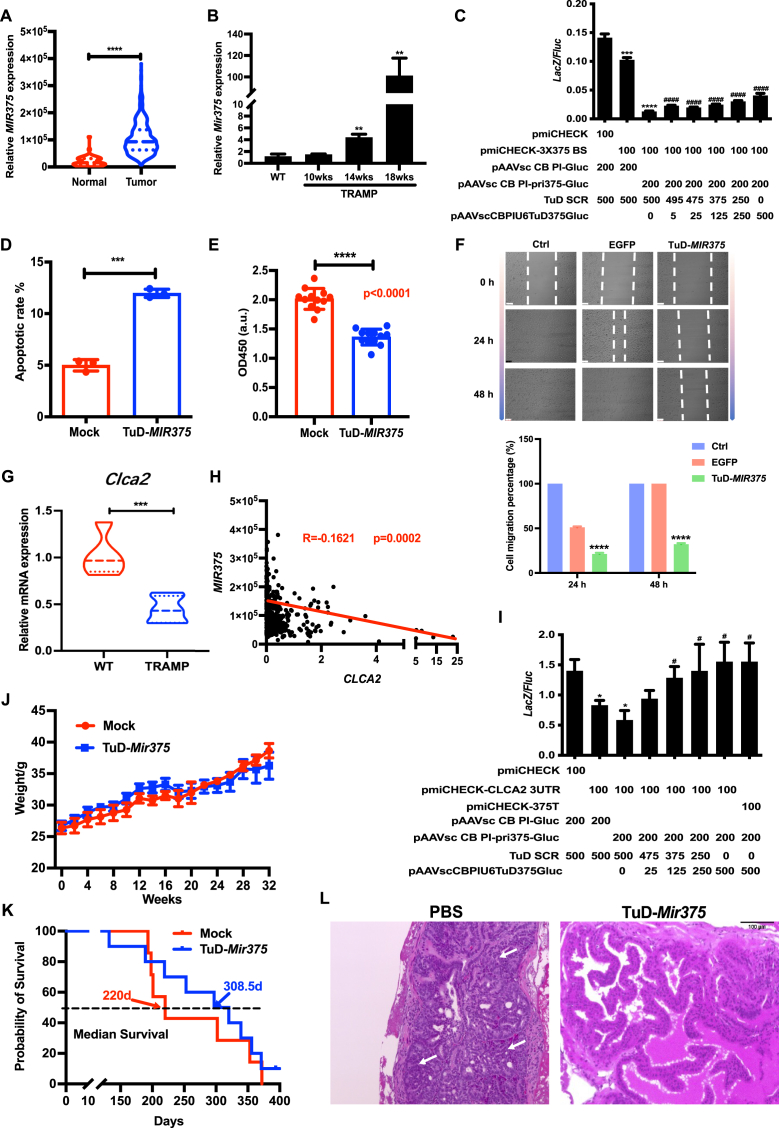
*MIR375* could inhibit apoptosis and promote proliferation and migration of prostate cancer cells via its target gene *CLCA2*, and rAAV9-TuD*-Mir375* improves the survival in TRAMP mice. **(A)** The expression level of *MIR375* in prostate cancer analyzed by the TCGA database. **(B)***Mir375* expression level evaluated by quantitative real-time PCR in WT and TRAMP mice at different ages. **(C)** TuD RNA was cloned into a lentiviral vector to inhibit *MIR375* function. The pmiCHECK plasmid DNA was used as the positive control, and the sequence of the triple *MIR375* binding site was inserted into the downstream region of the *LacZ* coding sequence (CDS) of pmiCHECK-3 × 375 BS. **(D)** Annexin V-FITC/PI staining analysis of PC3 cell apoptosis. **(E)** CCK8 assay was applied to detect cell viability. **(F)** Migration ability was assessed by wound-healing assay. **(G)***Clca2* expression between TRAMP mice and control mice. **(H)** Correlation analysis of *MIR375* and *CLCA2* expression. **(I)** TuD SCR partly reversed the *LacZ/Fluc* reporter activity after transfection with 3′UTR of *CLCA2*. **(J)** TRAMP mice were injected with rAAV9-TuD-*Mir375* or PBS, and mouse weight was recorded every two weeks. **(K)** The median survival time of mice injected with rAAV9-TuD-*Mir375* or PBS. **(L)** Prostates were assessed by hematoxylin and eosin staining. The white arrow reveals tumorigenesis. TRAMP, transgenic adenocarcinoma mouse prostate; WT, wild-type; TuD SCR, tough decoy-short consensus repeat; PBS, phosphate-buffered saline. ^∗^*P* < 0.05, ^∗∗^*P* < 0.01, ∗∗∗*P* < 0.001, ^#^*P* < 0.05, ^####^*P* < 0.0001; ∗and # indicate comparisons with the first column and the third, respectively.

Furthermore, we delved into the expression levels of *CLCA2* utilizing TCGA datasets, revealing a noteworthy decrease in *CLCA2* expression in PCa compared with normal tissues (Fig. S1C). Likewise, *Clca 2* expression level was significantly lower in TRAMP mice via performing quantitative real-time PCR of RNA extracted from TRAMP mice and control mice (Fig. 1G). Subsequently, we explored the potential relationship between *MIR375* and *CLCA2* in PCa. The *MIR375* expression was significantly negatively correlated with that of *CLCA2* by analyzing the expression data of the TCGA dataset (Fig. 1H). Additionally, *CLCA2* expression was increased through the inhibition of *MIR375* (Fig. S1D). Figure 1M shows the sequence alignment of *CLCA2* and the evolutionary conservation of 3UTR among different species. *CLCA2* 3UTR contained three possible *MIR375* target sites in the seed region (Fig. S1E). Figure S1F depicts the successful construction of the vector. We co-transfected *MIR375* with wild-type 3UTR of *CLCA2* or mutated 3UTR. Following transfection with the wild-type 3UTR of *CLCA2*, the *LacZ/Fluc* reporter activity was notably repressed, while this repression was diminished after transfection with the mutated 3UTR (Fig. S1G). Additionally, the *MIR375* inhibitor partially reversed the *LacZ/Fluc* reporter activity after transfection with 3UTR of *CLCA2* (Fig. 1I). These results indicated that *MIR375* can orchestrate *CLCA2* expression by binding to its 3′UTR sequence.

Importantly, we evaluated the therapeutic effect of injection of rAAV9 delivery TuD-*Mir375* in mice with PCa. After the rAAV9-TuD-*Mir375* injection, mouse weight was monitored every two weeks, and intriguingly, no substantial difference in body weight was discerned (Fig. 1J). Notably, the median survival time of mice in the AAV9-TuD-*Mir375* group (308.5 days) was significantly longer than that of mice in the phosphate-buffered saline (PBS) group (220 days) (Fig. 1K). More obvious tumorigenesis was observed in the PBS group than in the TuD-*Mir375* group by hematoxylin and eosin staining (Fig. 1L). The Preliminary experiments suggest that injection of rAAV9 delivery TuD-*Mir375* resulted in longer median survival times and less tumor angiogenesis in mice with PCa.

Our study unveiled that the expression of *MIR375* in tumor sites is significantly higher than that in normal tissue. Mainly as a tumor promoter, *MIR375* is reported to be associated with the prognosis of PCa patients.^4^ Specifically, apoptosis markers could be strongly inhibited by *MIR375* when the expression of downstream epithelial–mesenchymal transition and andrology receptor markers could be up-regulated.^2^ Consequently, *MIR375* emerges as a compelling candidate for novel therapeutic strategies in PCa patients. *In vitro* experiments were also conducted to systematically assess the impact of *MIR375* on the PCa cells. Our findings have elucidated that *MIR375* exerts a potent stimulatory effect on PCa progression by modulating the expression of downstream genes. Among these genes, *CLCA2* was identified as a potential target of *MIR375*. *CLCA2*, a member of the p53 family, is known for its capacity to suppress the proliferation, migration, and invasion of cancer cells.^5^ We found out that *MIR375* could orchestrate *CLCA2* expression by binding to its 3′UTR sequence when the expression of *CLCA2* was significantly negatively correlated with the *MIR375*. By this means, the inhibition of *MIR375* can activate the downstream tumor suppressor, *CLCA2*, thereby impeding the progression of PCa cells. Particularly, rAAV9 was proven effective in the transduction of target genes.^3^ The rAAV9-TuD-*Mir375* was constructed and intratumorally injected into the mice to further validate the effectiveness of AAV-based therapy. While no discernible difference in mouse weight was observed between the two groups, the rAAV9-TuD-*Mir375* group exhibited a significantly prolonged survival time in comparison to the PBS group. Apart from that, the tumors in both groups were retrieved to perform hematoxylin and eosin staining. Similarly, the tumorigenesis was decreased after the delivery of TuD-*Mir375* compared with the PBS group. Thus, the *in vivo* experiments have corroborated that the down-regulation of *Mir375* holds promise for enhancing the survival of TRAMP mice.

In conclusion, targeting *MIR375* may effectively down-regulate the proliferation and invasion of PCa cells, and rAAV9 was a robust tool for the transduction of genes into target cells or organs. These findings may pave an avenue for the genetic therapy of PCa, which constitutes the comprehensive treatments and management of PCa.

## Ethics declaration

The animal study was reviewed and approved by the Animal Ethics Review Committees of the West China Hospital.

## Author contributions

JA and XY conceived the project and drafted the manuscript. JL, XY, ZH, and DL performed the experiments. TZ and JY collected the public data and performed the analysis. JA revised the manuscript. All authors contributed to the article and approved the submitted version.

## Conflict of interests

The authors declare no competing interests.

## Funding

This study was supported by the National Key R&D Plan (2023YFC3403200), grants from the 10.13039/501100001809National Natural Science Foundation of China (No. 82070784 and 81702536) and a grant from the Science & Technology Department of Sichuan Province, China (No. 2022JDRC0040).
