# Supplementary material for: Adeno-associated virus-mediated intraprostatic suppression of MIR375 inhibits tumor progression in the TRAMP mouse model of prostate cancer
Source: Genes Dis. 2023 Nov 23;11(6):101182. doi: 10.1016/j.gendis.2023.101182 (PMC11298863; doi:10.1016/j.gendis.2023.101182)
Supplement: Multimedia component 1 [file mmc1.docx]

**Title: AAV-mediated intraprostatic suppression of *MIR375* inhibits tumor progression in the TRAMP mouse model of prostate cancer**

**Author:** Xianyanling Yi^1#^, Jin Li^1#^, Zeyu Han^1#^, Tianyi Zhang^1^, Dazhou Liao^1^, Jia You^1^, Jianzhong Ai^1*^

**Affiliation:** ^1^Department of Urology/Institute of Urology, West China Hospital, Sichuan University, 88 South Keyuan Road, Chengdu, 610041, China.

^#^These authors contributed equally as the first author.

***Correspondence to**: Jianzhong Ai at Department of Urology, Institute of Urology, West China Hospital, Sichuan University, 88 South Keyuan Road, Chengdu, 610041, P. R. China. E-mail address: jianzhong.ai@scu.edu.cn (J. Ai).

**Supplementary methods/materials:**

**This supplementary file shows the methods applied in supporting the results in the main text.**

**Genomic data source and preprocessing**

PRAD mRNA and miRNA expression data were downloaded from The Cancer Genome Atlas (TCGA) database (http://cancergenome.nih. gov/). The *MIR375* expressions were normalized by reads per million mapped reads (RPM) values (RPM = (number of reads mapping to miRNA/number of reads in clean data) × 10^6^). mRNA expressions were calculated by fragments per kilobase of transcript per million mapped reads (FPKM). ID conversion file (gencode.v38.annotation.gtf) was used for annotation of gene IDs. Differential expression analysis was performed to compare the *MIR375* and *CLCA2* differentially expressed between tumor and adjacent normal tissue. Spearmans correlation analysis was conducted for correlation of *MIR375* and *CLCA2* expression.

**TRAMP mouse model and cell culture**

Wild-type (WT) C57BL/6 mice and transgenic adenocarcinoma mouse prostate (TRAMP) model were acquired from the Jackson Laboratory^1,2^. Mice were maintained in standard conditions and grouped randomly. The animal study was reviewed and approved by the Animal Ethics Review Committees of the West China Hospital. All cells were obtained from ATCC (Manassas VA). PC3 cells were cultured with RPMI 1640 medium, while TRAMP-C2 and 293 cells were cultured with DMEM in a humidified atmosphere of 5% CO2 at 37 °C. The culture media were supplemented with 10% fetal bovine serum (FBS) and 1% penicillin-streptomycin.

**Quantitative real-time polymerase chain reaction**

RNA was extracted by using TRIzol reagent (Invitrogen) and then reverse-transcribed into cDNA using a Transcriptor First Strand cDNA Synthesis kit (Thermo Fisher, Cat. No. K1622). Nanodrop 2000 (Thermo Fisher) was used to measure the concentration of RNA. Quantitative RT‒PCR was performed using QuantiNova SYBR Green PCR Kit (QIAGEN, Cat. No. 208054). The *β-Actin* gene was used to normalize the expression of various genes. The primers used to detect mRNA levels are listed Table S1.

**Dual-reporter gene assay**

The expression of the *MIR375*-target gene was verified in 293 cells by dual reporter gene assay. The pmiCHECK vector which harbored β-galactosidase gene z (*LacZ*) and firefly luciferase (*Fluc*) was constructed, and 3×*MIR375* binding sites were inserted into the downstream of the *lacZ* gene open reading frame (ORF). The plasmid pAAVsc CB PI-pri375-Gluc was generated by inserting the sequence of pri-*MIR375* into the multicloning site of the pmiCHECK. Tough decoy (TuD) RNA is the inhibitor that target specific miRNAs efficient. We used TuD-short consensus repeats (SCRs) cloned into lentiviral vector to inhibit *MIR375* function. Then, the indicated plasmids were co-transfected into HEK-293 cells. Forty-eight hours post-transfection, the expression of the *LacZ* and *Fluc* reporter gene activity was assessed by a Galacton-Star kit (Thermo Fisher, Cat. No. T2265) and the Luciferase Reporter Assay System (Promega, Cat. No. E1500).

Reporter gene assay was also applied to validate *CLCA2* as *MIR375* target genes. The 3′-UTR and mutant 3′-UTR of *CLCA2* was cloned into pmiCHECK vector to build wild type (WT) and mutant type (MT) of *CLCA2* 3′-UTR luciferase report vectors. The 293 cells were co-transfected with the 3′-UTR *CLCA2*, *MIR375* or *MIR375* inhibitor. After 48 hours of transfection, the activities of *LacZ* and *Fluc* were assessed.

**Apoptosis assay**

Cell apoptosis was conducted using Annexin V-FITC/PI cell apoptosis detection kit (4A Biotech, Cat. No. FXP018-100). Cells were harvested and washed with PBS for three times, and stained with binding buffer, fluorescein isothiocyanate (FITC)-annexin V and propidium iodide (PI) staining, then incubated for 10 min s at room temperature. Flow cytometry data were collected on Guava easyCyte Flow Cytometer (Millipore Sigma) and further analyzed by Flow Jo software (V10).

**Cell counting kit-8 (CCK8) assay**

The CCK8 assay was performed using CCK8 kit (Abmole, Cat. No.M4839) to detect cell proliferation capacity. Cells were planted in 96-well plates and cultured for 48 hours after transfected plasmids expressing green fluorescence protein and TuD-*MIR375*. Then 10 μL of CCK8 was added to each well, and cells were incubated at 37°C in the dark. After 2 hours, the cell proliferation ability was assessed at 450 nm.

**Wound healing assay**

In order to assess the effect of *MIR375* on the cell motility properties of PCa cell, wound healing assay was performed. One day before transfection, PC3 cells were paved on six-well plate. Then PC3 cells were transfected with the TuD-*MIR375* plasmid for 48 h. When the cell confluence was about 95%-100%, vertical lines were drawn in the culture wells with 10µL pipette tip, and each scratch should be roughly the same width. The floating cells were washed off using PBS, and medium containing 1% FBS was used to culture the cells. Images were captured with a Zeiss inverted microscope after 24 and 48 h.

**Intratumoral injection of** **TRAMP mice**

For rAAV9 can effectively and safely transduce mouse prostate *in vivo*, AAV9-TuD-*Mir375* was constructed as previously described^3^. Viruses were purified with cesium chloride (CsCl) gradient and titered by silver staining and qPCR^4^. 6 weeks old male C57BL/6 mice were selected for our experiment, and they were randomly arranged to two groups (*n*=5). 3×10^6^ TRAMP-C2 cells in 100µL PBS were injected subcutaneously into the right flanks of the mice. When the tumor size was about 100mm^3^, we intratumorally injected AAV9-TuD-*Mir375*, and the control group was injected with an equivalent volume of sterile PBS. The body weight was measured at two weeks intervals. The survival status of the two groups was recorded and the Kaplan-Meier (KM) method is used to analyze the survival situation.

**HE staining analysis**

The mice were sacrificed and tumor tissue was collected for histological analysis by hematoxylin and eosin (HE) staining. Briefly, tissue sections were fixed in 10% buffered formalin at room temperature overnight, and then sectioned to a thickness of 4µm after having been embedded in paraffin. HE staining was performed using an HE staining kit (Solarbio, Cat. No. G1120) according to standard protocols.

**Statistical analysis**

All experimental results were presented as the mean ± standard deviation (SD). Data were analyzed with GraphPad Prism version 8.0 (GraphPad Software). Differences between groups were evaluated using the student *t* test. *Spearman* correlation test was applied to determine the correlation between expression of *MIR375* and *CLCA2*. Survival analysis was performed by the KM method. Statistical significance was set to *p* < 0.05.

**Reference:**

1. Huss WJ, Maddison LA, Greenberg NM. Autochthonous mouse models for prostate cancer: past, present and future. *Seminars in Cancer Biology*. Jun 2001;11(3):245-259. doi:10.1006/scbi.2001.0373

2. Greenberg NM, Demayo F, Finegold MJ, et al. PROSTATE-CANCER IN A TRANSGENIC MOUSE. *Proceedings of the National Academy of Sciences of the United States of America*. Apr 1995;92(8):3439-3443. doi:10.1073/pnas.92.8.3439

3. Sena-Esteves M, Gao G. Introducing Genes into Mammalian Cells: Viral Vectors. *Cold Spring Harbor protocols*. 2020 08 2020;2020(8):095513. doi:10.1101/pdb.top095513

4. Ai J, Li J, Gessler DJ, et al. Adeno-associated virus serotype rh.10 displays strong muscle tropism following intraperitoneal delivery. *Sci Rep*. Jan 9 2017;7:40336. doi:10.1038/srep40336

**Table S1 Primer sequences for qPCR.**

| *CLCA2* | Forward (5′-3′) | CATCGGGCTGAAACTTGTGAC |
| --- | --- | --- |
|  | Reverse (5′-3′) | TCTTGGTCGCATTGAACAGGT |
| *β-actin* | Forward (5′-3′) | GTGACGTTGACATCCGTAAAGA |
|  | Reverse (5′-3′) | GCCGGACTCATCGTACTCC |

**Supplementary figure:**

(A) Dual-gene reporter vector was constructed to assess the activity of *MIR375*. The pmiCHECK plasmid DNA was used as the positive control, and the sequence of the triple *MIR375* binding site was inserted into the downstream region of the LacZ coding sequence (CDS) of pmiCHECK-3×375 BS. (B) Annexin V-FITC/PI staining analysis of PC3 cell apoptosis. (C) *CLCA2* expression between tumor and normal tissues using TCGA database. (D) The relative expression levels of *CLCA2* after *MIR375* inhibition. (E) Alignments of *CLCA2* and the 3UTR from human, rhesus, mouse, dog, elephant, and the target seed region of *MIR375* in the 3UTR region of *CLCA2*. The red line represents the target seed region. (F) *CLCA2* was cloned into pmiCHECK vector, and after increasing the *MIR375* expression, *LacZ/Fluc* gene expression was weakened. (G) The LacZ/Fluc reporter activity was decreased by transfecting with wild-type 3UTR of *CLCA2*, and transfection with mutated 3UTR had no effect on the *LacZ/Fluc* activity. TuD SCR, tough decoy- short consensus repeat. ∗p < 0.05, ∗∗p < 0.01, ∗∗∗p < 0.001, ∗∗∗∗p < 0.001.
